# Microstructural Stability and Transition to Unstable Friction for FCC Metals: Ag and Ni

**DOI:** 10.3390/ma18174123

**Published:** 2025-09-02

**Authors:** Alexey Moshkovich, Inna Popov, Sergei Remennik, Lev S. Rapoport

**Affiliations:** 1 Department of Science, Holon Institute of Technology, Holon 5810201, Israel; alexeym@hit.ac.il; 2 Center for Nanoscience and Nanotechnology, Hebrew University of Jerusalem, Jerusalem 91904, Israel; innap@savion.huji.ac.il (I.P.); sergeir@savion.huji.ac.il (S.R.)

**Keywords:** dislocations, friction, wear, ductility, brittleness, segregation

## Abstract

The effect of dislocation pile-ups responsible for the generation or annihilation of dislocations during friction of Ag and Ni was considered. The steady-state friction was accompanied by the formation of twin bundles, intersecting twins, dislocations, adiabatic elongated shear bands, and intense dynamic recrystallization. The mechanisms of microstructural stability and friction instability were analyzed. The theoretical models of dislocation generation and annihilation in nanocrystalline FCC metals in the context of plastic deformation and failure development under friction were proposed. The transition to unstable friction was estimated. The damage of Ag was exhibited in the formation of pores, reducing the contact area and significantly increasing the shear stress. The brittle fracture of Ni represents a catastrophic failure associated with the formation of super-hard nickel oxide. Deformation resistance of the dislocation structures in the mesoscale and macroscale was compared. The coefficient of similitude (K) has been introduced in this work to compare plastic deformation at different scales. The model of the strength–ductility trade-off and microstructural instability is considered. The interaction between the migration of dislocation pile-ups and the driving forces applied to the grain boundaries was estimated. Nanostructure stabilization through the addition of a polycrystalline element (solute) to the crystal interiors in order to reduce the free energy of grain boundary interfaces was investigated. The thermodynamic driving force and kinetic energy barrier involved in strengthening, brittleness, or annealing under plastic deformation and phase formation in alloys and composite materials were examined.

## 1. Introduction

In this Introduction, we will briefly consider the main trends in the study of the deformed structure surface layers under friction in the steady state and the transition to unstable friction. As such, thermo-mechanical and structural analyses of contact interaction will be evaluated. Recently, the mechanical properties of surface layers were improved by the development of gradient nanocrystalline layers [1]. A stable gradient nanograined surface layer significantly reduces the coefficient of friction (COF) of a Cu alloy under high-load dry sliding [2]. Subsequent studies showed that grain refinement occurs after several sliding passes during the running-in of Cu [3]. The dominant mechanisms in the steady friction state are the hardening and softening of surface layers [4]. Moreover, dynamic plastic deformation at cryogenic temperatures was studied [5]. The dislocation manipulation and rearrangement, deformation twinning forming, nanoscale twin/matrix (T/M) lamellae in bundles, and shear banding were identified as dominant mechanisms of DPD. Plastic deformation via shearing leads to the formation of the ultrafine-grain structure observed in severe plastic deformation processes [6]. Grain boundary (GB) stabilization and ultrahigh hardness can be explained by sliding-induced strain localization and microstructural instabilities [7]. In further developments associated with gradient nanostructures, it was shown that gradient nanostructured surface layers of steel exhibit low COFs and enhanced wear resistance [8]. The enhancement of wear resistance was attributed to the high strain accommodation capacity and high hardness of the material. A relatively new method for improving strength and ductility is the creation of heterogeneous nanostructures in metals [9]. High entropy alloys exhibit the synergistic effects of multiscale precipitates, improve resistance to shear deformation, and facilitate the formation of a dense, stable oxide film that effectively decreases wear damage [10,11].

The microstructure evolution and deformation mechanisms underlying the formation of dislocation structures after the first loading steps have been discussed [12]. The effect of sliding direction and grain orientation on crystal rotation accommodation was analyzed [13]. Continuum modeling of dislocation microstructures under friction loading was proposed [14]. A crystal plasticity model of micro- and macro-scale plastic deformation under friction conditions was analyzed. The influences of plastic deformation, surface topography, and contact area evolution were estimated. Summarizing the analysis of deformed structure under friction and wear, we can note the intensive investigation of plastic deformation of surface layers under friction at different length scales. However, our understanding of the interaction between external parameters of loading and internal structural parameters is not yet so clear.

Plastic deformation of surface layers is mainly determined by the stacking fault energy (SFE) (e.g., [15,16,17,18]) deformation twinning [19,20,21]. The substantial role of the stacking fault energy (SFE) of fully nano-twinned Cu alloys [22] and brass [23] was discussed. Pure Cu, Cu-10 wt.% Zn, and Cu-30 wt.% Zn exhibited average grain sizes of approximately 75 nm, 50 nm, and 10 nm, respectively, under hot-pressing torsion (HPT) conditions [15,18]. Moreover, it was shown that the densities of dislocations and twins increased with increasing Zn content and thus decreased with the SFE. It was demonstrated that after HPT of Cu-30 wt.% Zn, equiaxed ultrafine-grained (UFG) crystals are divided into twin lamellae. [16]. Gradient nanograined–nano-twinned layers in Cu-4.5% Al alloy were recently analyzed using an ultra-precision machining technique [24]. The low COFs observed under friction were attributed to grain refinement, grain coarsening, and dynamic recrystallization. Moreover, the mechanical and structural stabilities of the gradient nano-twinned structure were attributed to twin boundary migration and grain rotation. An ideal critical twin boundary spacing was proposed for nano-twinned metals based on molecular simulations [25].

The correlation between grain orientation in a polycrystal and the deformation microstructure was analyzed using a scaling hypothesis and the principle of similitude [26]. The unified ductility equation, which defines the relationship between the tensile behavior of Cu and specimen dimensions, was considered [27]. Ductility equations obtained at the mesoscale and macroscale levels were compared. All analyzed processes, such as hardening, dynamic recovery, and dynamic recrystallization, are strongly dependent on the stability of grain boundaries [28,29,30,31,32]. The effects of grain size on the cyclic deformation of nickel have been studied [33]. The stresses were separated into back stress and effective stress; moreover, the back stress was divided into intragranular and intergranular interactions. The concept of grain boundary sources and sinks in the deformation of nanocrystalline metals was identified in molecular dynamics simulations [34]. The total migration of a boundary consists of both translations approximately normal to the boundary and lateral changes in area.

Enhancing the strength of metals and alloys while maintaining their ductility has been a long-standing challenge [35,36,37,38,39]. The low ductility of nanocrystalline (NC) metals leads to plastic instabilities, accompanied by shear banding, development of necking, and catastrophic failure [35]. At the same time, cold-drawn metals lack high strain hardening. Most mechanisms of plastic deformation provide high strength; however, they lead to a loss of ductility; this effect is called the strength–ductility trade-off [40,41]. To enhance both strength and ductility, high-entropy alloys (HEAs) with phase-stabilization microstructures were designed. The hardening of Hadfield manganese steel and high-strength Fe–Mn-(Al, Si) twinning-induced plasticity (TWIP) steels was improved due to the decrease in phase stability [42,43,44]. It was demonstrated that introducing a definite sum of plasticizing structures with remarkable work-hardening properties is key to enhancing the strength–ductility of TiAl. The authors started with the important remark that low ductility is an “Achilles’ heel” of high-strength materials [45]. The gradient nanostructured metals and alloys with improved strength–ductility synergy and hardenability were obtained through grain refinement, distribution, and transformation of phase or chemical elements [46,47]. Meanwhile, the analysis of the factors responsible for high mechanical properties does not explain the onset of necking under tension, fatigue, or friction. Various fundamental questions regarding the enhancement of strength, ductility, and handleability of different metals and alloys, as well as methods of hardening/softening, are still being debated and intensively investigated, and successful phenomenological models of the interaction between externally applied and internal shear stress in grain boundary microstructures remain unclear.

The purpose of this study is to develop a crystal plasticity constitutive model for describing the plastic deformation of face-centered cubic (FCC) metals under steady-state friction, microstructural stability, and instability transitions. A thermo-kinetic approach was used to analyze microstructural stability and instability. Section 2 describes the microstructural stability of FCC metals and alloys. The materials and methods used in the friction tests, as well as the characterization of the deformed structure of the surface layers, are presented in Section 3. Section 4 explores the balance between work hardening and softening, driven by deformation twinning, shear banding, and dynamic recovery during friction between Ag and Ni. In Section 5, microstructural instability under Ag and Ni friction is investigated. The mechanisms of ductile failure of Ag under friction are compared with the brittle fracture of Ni. Then we focused on the theoretical analysis of the strength–ductility trade-off model and the transition to instability based on an analysis of thermodynamic approaches. This crystal plasticity model of microstructural stability and instability is proposed to offer new insights into the mechanisms of friction and wear in FCC metals and alloys.

## 2. Theoretical Background: Microstructural Stability of FCC Metals and Alloys

**Figure 1:**
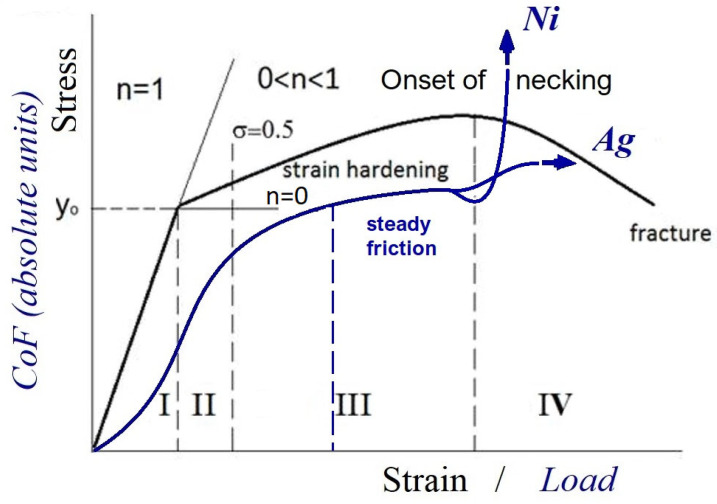
The original stress–strain curve of monotonic/cycling phases of loading, and the original curve of the coefficient of friction (COF) (σ is stress and n is the strain hardening exponent).

We consider in the ensuing discussion the parameters responsible for the microstructural stability of FCC metals and alloys under plastic deformation under different loading conditions. We begin with the stress–strain loading curve (Figure 1).

The first and second stages of stress–strain loading in the friction process correspond to the running-in phase, characterized by dislocation intersection and grain boundary shearing due to the dislocation pile-up (stages I and II in monotonic or cyclic loading). A steady friction state corresponds to stages III and IV, where strong grain boundary shearing and high dislocation density result in high strength and relatively low ductility [48,49,50,51,52]. At large strains (stage IV), work hardening is characterized by an equilibrium between dislocation accumulation and dynamic recovery [53,54,55,56,57]. The balance between the generation and annihilation of dislocations was captured by Pantleon in his exemplary work [58]. It is noted that the storage and annihilation of dislocations proposed by Kocks [59] does not differentiate between dislocations of opposite signs and neglects the possible existence of an excess of dislocations of one sign. Pantleon reformulated the balance equation for the dislocations of opposite signs to the total dislocation flux (j).

(1) $$j=j\to +j\leftarrow =\frac{\dot{\gamma }}{b},$$

where $\dot{\gamma }$ is the strain rate and b is the Burger’s vector. The balance equation of Kock’s model is given as follows:

(2) $$\frac{d\rho }{d\gamma }=\frac{1}{\lambda_{p}b}-\frac{2y}{b\rho },$$

where *λ_p_* is the mean free path and 2*y* is the dislocation length. The rate of dislocation presented by the authors includes a third term, leading to an increase in the total dislocation density without variations in the storage mechanisms; i.e., disorientation can occur in the slip system from both sides of the grain boundary.

(3) $$\frac{d\rho }{dt}=\left(\frac{1}{\lambda }-2y\rho \right)j+2y∆\rho ∆j$$

In this study, we will adopt Pantleon’s idea to better understand the effect of nanocrystalline grain boundaries on the hardening–softening phenomena. Further, necking, similar to the onset of strong failure (ductile shearing through softening or brittle fracture after critical hardening), occurs. In this instance, the possibility of varying the activation barrier via strengthening or softening through thermo-mechanical and chemical interactions at grain boundaries can be useful in designing friction and wear behavior. We hope to analyze this in the theoretical section.

Ultra-fine grain (UFG) metals and alloys typically exhibit high strength and ductility and are widely used in different engineering applications, including those involving fatigue and friction. Applying high loads and temperatures significantly increases the longevity of the contact surfaces. In this instance, nanocrystalline grain boundaries (GBs) are gaining importance. The mechanical behavior of nanocrystalline metals and alloys has been studied under severe contact conditions of fatigue and fracture [60]. The effect of a pile-up of dislocations at UFG boundaries responsible for the generation or annihilation of dislocations was considered. It is also noteworthy that stress relaxation at the grain boundary, leading to annealing, can increase the strength of metals and alloys; moreover, atomic shuffling can play an important role in atomic motion at grain boundaries [60,61]. It is also vital to preserve a stable nanocrystalline structure during severe plastic deformation in the steady state and to suppress the transition to instability [62]. In the transition to unstable friction, the grain boundary energy is increased significantly, leading to high COF and temperature. Grain coarsening under the deformation of Cu at cryogenic temperatures occurs much faster than at room temperature; moreover, it has been shown that the deformation mechanisms under SPD of NC materials are mainly determined by the formation of deformation twins and stacking faults. The analysis of deformation twinning processes, constitutive modeling, and simulations of NC materials at different length scales was recommended for a better understanding of the mechanical properties of NC materials. Here, it is worth noting that studied ultrathin foils of a few grains can be distorted by an electron beam by enhancing dislocation motion and diffusion activity. This means that the variation in average nanograin size by nanometers cannot reflect bulk deformation properties [62].

It is important to emphasize that the stabilization of the nanocrystalline GS can be achieved through solute segregation, especially at high temperatures. The most significant solute effects are achieved by reducing GB energy [63,64,65]. The thermodynamic and atomistic simulations demonstrate a strong influence of Ta on segregation in GBs, triple joints, and stacking faults of copper [66]. The mechanisms responsible for the stability of Cu-10% Ta alloy are based on thermodynamic and kinetic approaches. Vickers microhardness of nanocrystalline Cu–10 at. % Ta after milling was two times higher than that of pure nanocrystalline Cu (5 GPa and 2.5 GPa, respectively). Furthermore, the stability of nanostructured tungsten alloys was estimated based on the development of the map [67]. The stability of the W-20 at. % Ti alloy with an average grain size of approximately 20 nm was analyzed. Nanostructure stabilization involves adding a polycrystalline element (solute) to the crystal interiors to reduce the free energy of grain boundary interfaces; specifically, the segregation state at grain boundaries should provide a global minimum on such surfaces. It is worth noting that a stable grain size of approximately 20 nm was preserved under annealing at 1000 °C, whereas strong instability was evident for unalloyed tungsten. Recently, the microstructural stability of nickel-based (Al:Nb ratio) superalloys was studied at 700 °C over a long exposure period [68]. The strengthening of different precipitations was estimated in the analysis of strong or weak-coupled dislocation pairs and the dispersion of tetragonally distorted particles. Further refinement of complex systems is key to the development of new strengthening materials. One of the early simulations of the stabilization of nanocrystalline materials was performed using dopants in copper [69]. The calculated energy of the grain boundary decreased to zero with increasing dopant coverage. However, all the dopants are limited to a small region of approximately one nanometer along the grain boundaries. The thermo-mechanical properties of the Al-Ni-Y system were optimized by minimizing the total amount of alloying [70]. Co-segregation of the Ni and Y atoms at the grain boundaries is accompanied by the formation of nanocrystalline grains approximately 14 nm in size. The influence of all compositions of Al-Ni-Y on the relative hardness (ΔH/H_0_) and its retention at definite temperatures was estimated. The hardness of all compositions decreased by up to 40% above a margin temperature. At a specific temperature, the hardness of the composition dropped below ΔH/H_0_ < 0. This temperature was denoted as the “retention temperature”. Here, we present the annealing of the Al-Ni-Y system, which leads to grain growth and hardness reduction that correlates with the formation of intermetallics. Then, the microstructural stability at high temperature and the enhanced hardness of co-segregated compositions were established.

The mechanisms of microstructural stabilization were also investigated on an atomic scale [71]. The simulation of the deformed structures and mechanical properties of nanocrystalline materials requires our understanding of the temporal and spatial scales in the analysis of plastic deformation. Consequently, inter-grain deformation occurs below a critical grain size (<10–12 nm), while with further increases in grain size, the dominant mechanisms are varied to intra-grain, accompanied by the emission of dislocations from the grain boundaries. Moreover, atomic motion is characterized by atomic shuffling, indicating a shift of an atom from one grain to a neighboring one. The atomistic mechanism of strain localization was studied using MD simulations of nanocrystalline Ni [72]. The effect of strain and sample size on strain localization was estimated. Strain localization is mainly determined by grain rotation at small grain sizes. It is of interest that a highly localized strain (~18%) of Ni was accompanied by remarkable grain coarsening. The dominant mechanisms of this grain growth may be the migration of grain boundaries and the connection of grains due to their rotation. A method for suppressing strain localization was also proposed: selective doping for grain resistance, a technique widely developed and used in many of the subsequent studies.

We now turn from the process of microstructural stabilization to the processes responsible for the transition to instability due to the application of segregation engineering. In our opinion, these processes can be described using general constitutive models of crystal plasticity of FCC metals and alloys. In recent years, segregation engineering has been widely used for the characterization of thermal and mechanical stability of composite materials and evaluation of microstructural transitions to instability. Understanding the evolution of grain boundaries and grain boundary precipitation in alloys and composite materials is crucial in the development of new materials with high resistance to temperature and contact loads under tensile strength [73,74], fatigue [75], friction [76], and corrosion tests [77]. Grain boundary engineering was based on the potential of weakening or strengthening grain boundaries (GBs) [78,79,80]. The solute decoration and confined transformation in grain boundary microstructure are referred to as “grain boundary segregation engineering” (GBSE) or “segregation engineering” [81]. Segregation engineering is applied today to strengthen alloys and composite materials, increase or decrease the average grain size, and increase the stability of materials under high temperatures and loads in different engineering applications. Analyzing deformed structures is challenging because GB segregation processes occur at different times and length scales. The modern methods for characterizing GB segregation were represented in [82]. The effect of solute segregation on the variation of cohesive energy at the grain boundary of pure metals leading to brittle fracture was analyzed [83]. The developed GB cohesion maps indicate the influence of solute–solvent pairs on weakening or strengthening of grain boundaries. Brittleness occurs when a solute has a lower cohesive energy at the boundary and segregates in the first place. A positive grain boundary cohesion energy represents a net increase in the thermodynamic resistance to decohesion, while negative energy represents a tendency for embrittlement.

It is important to emphasize that optimal mechanical properties of microstructures are determined by phase transformations (PTs) and plastic deformations (PDs), which are responsible for the strength–ductility trade-off between the chemical driving force, ΔG_chem;_ strain energy, ΔG_strain_, and interface energy, ΔG_inter_ [84]. Generally, the phase transformation can lead to a decrease in the driving force ΔG and an increase in the kinetic energy barrier Q, thus providing a stable state. It has been confirmed that segregation plays an important role in grain boundary precipitation. Further analysis of segregation in grain boundaries will be considered in the theoretical section of the study. Furthermore, thermodynamic and kinetic approaches have been used to enhance resistance to grain coarsening in nanocrystalline Al-Mg alloys [85]. It was shown that the coarsening resistance is attributed to the segregation of Mg in grain boundaries and the formation of nanoscale Al_3_Mg_2_ precipitates. Hence, thermodynamic contributions and kinetic barriers to grain coarsening are the driving forces for nanocrystalline stabilization in Al-Mg alloys at high temperatures. A similar effect of surface segregation on the wear resistance of self-adaptive multi-principal element alloys (MPEAs), such as CrCoNi, was recently investigated [76]. It was found that high wear resistance is associated with the redistribution of nickel on the surface layers of CrCoNi, with the formation of amorphous-crystalline oxides in the top surface layers. Significant segregation of Ni in the first adhesive layer (~45%) was observed. It is suggested that Ni segregation is driven by thermodynamic forces rather than environmental effects; moreover, it is believed that kinetic forces play an important role in the redistribution of Ni in the CrCoNi alloy. A fundamental rigorous analysis of grain boundary segregations based on the study of the competition between chemical and elastic energies in the segregation process was recently presented [74]. The segregation energy, E_seg_ = E_segchem_ + E_segelas_, includes chemical and elastic contributions, respectively. Since E_elas_ and E_seg_ can be opposite to E_str_ (positive or negative), different effects on GB segregation can appear: a more positive (negative) E_seg_ may correspond to a more negative (positive) E_str_, showing a weaker (stronger) stabilizing effect but a stronger (weaker) strengthening effect. The authors constructed the maps of strengthening energy and GB segregation energy, indicating a trade-off relationship.

Summarizing the analysis of the microstructural stability of FCC metals and alloys, we conclude that the models discussed above should be applied to analyze shearing in surface layers under contact interaction and relaxation events occurring under strain localization during friction. To the best of our knowledge, the mechanisms of the microstructural stability and friction instability, the theoretical model of dislocation generation, and the annihilation in ultrafine-grained (UFG) or nanocrystalline (NC) FCC materials have not been systematically investigated, particularly in the context of plastic deformation and failure development under friction. Microstructural stability and transition to instability will be carefully considered. Thermodynamic driving forces and kinetic energy barriers in the generation of strengthening, brittleness, or annealing under plastic deformation or phase formation in alloys and composite materials will be analyzed. The application of grain boundary engineering in the processes of hardening/softening–brittleness will also be considered. The model of the strength–ductility trade-off with application to friction will be highlighted.

## 3. Materials and Methods

### 3.1. Friction and Wear of Ag and Ni

All friction tests were conducted under laboratory conditions (temperature, T = 25 °C, humidity ~50%) using a homemade pin-on disk rig, as described in our previous studies [86,87]. The running-in process occurred at a load of 8 N and continued for approximately 150 min, until nearly 90% of the surface of the pin was in contact. The sliding velocity was constant, at 0.37 m/s (300 rpm). The average temperature near the contact interface under steady friction was about 80 °C. This temperature is less than 0.1 of the melting temperature of silver, and, therefore, the temperature effect was excluded in the analysis of plastic deformation in a steady state.

Some parameters, such as the shear modulus (G), Burger’s vector (b), stacking fault energy (γ_SF_), virgin hardness (H_i_), and after friction (H_f_) and of the studied materials are presented in Table 1 for the following analysis of the results.

### 3.2. Characterization Methods

Microhardness was measured before and after friction tests at a load of 0.1 N for 10 s. The wear surfaces were carefully examined using a field-emission scanning electron microscope (SEM). The deformed microstructure of Ag after friction in the steady state and after the transition to an unstable state was analyzed using SEM, TEM, CTEM, and selected-area diffraction (SAD) patterns. The cross-section was prepared with a Helios Nanolab 460 F1 Lite Dual Beam Focused Ion Beam (DB-FIB) Scanning Electron Microscope (SEM) operated with Ga+ ions and a Thermo Fisher Scientific (TFS) (Waltham, MA, USA) Gas Injection System (GIS) operated with carbon and platinum. Transmission Electron Microscope (TEM), Scanning Transmission Electron Microscope (STEM), and Energy-Dispersive X-ray Spectroscopy (EDS) analyses were conducted using an Aberration Prob-Corrected S/TEM Themis Z G3 (TFS) operated at 300 KV and equipped with a Ceta camera, a high-angle annular dark field detector (Fischione Instruments, Export, PA, USA), DF/BF detectors, and a Super-X EDS detection system (TFS).

### 3.3. Analysis of Plastic Deformation of Ag and Ni in a Steady Friction State

**Figure 2:**
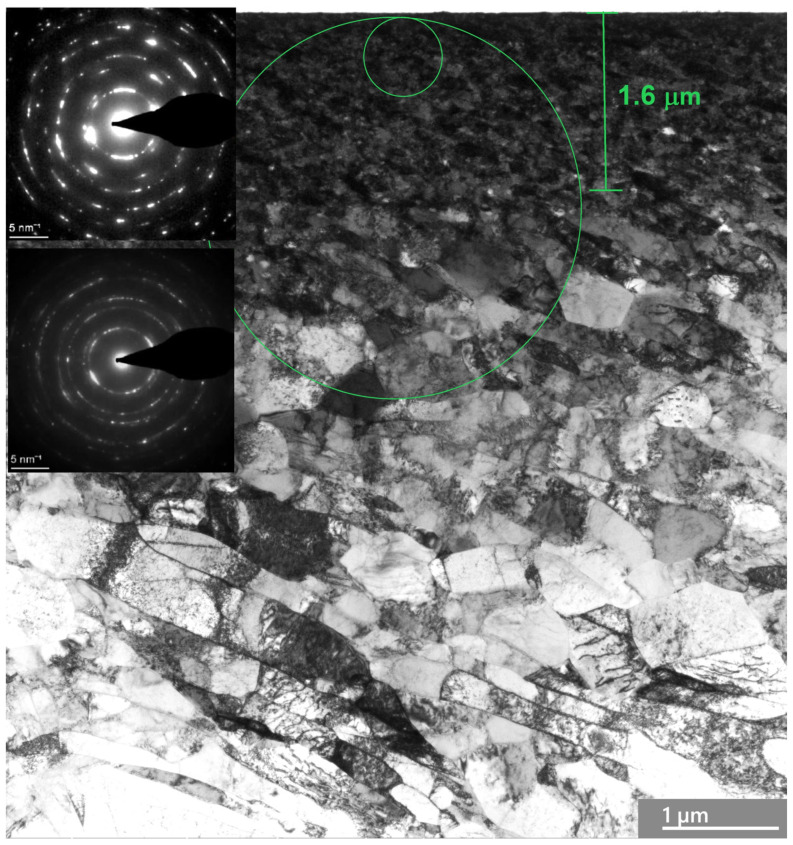
TEM bright-field image and diffraction patterns taken from the surface layers of Ag after friction in the steady state. (The top surface indicates a nanocrystalline structure with pores, and sublayers are characterized by the shear band inclined about 40° to the friction surface). The upper diffraction pattern taken from the small green circle corresponds to this area. The insert of diffraction in the middle of the image represents diffraction in the big green circle area.

To characterize the deformed microstructure of Ag, the generation and annihilation of dislocations and the effects of twins and adiabatic shear banding on friction in the steady state and in the transition to instability are analyzed. A strong gradient nanostructure in the surface layers of Ag after steady-state friction is depicted in Figure 2. TEM observations show a gradient nanograined structure, with the top surface layer consisting of nearly equiaxed ultra-fine grains, having an average transversal grain size of ~33 nm and a thickness of ~1.6 μm. The image demonstrates the ultra-fine top surface layer of Ag. The SA diffraction corresponding to the outermost 380 nm of the surface layer demonstrates a fine mosaic structure with highly misoriented domains of the original grains. These nanocrystalline grains are highly defective, similar to those observed in nanostructured Cu prepared using other top-down approaches, such as dynamic plastic deformation [16,17,18].

The sublayers represent large, randomly oriented grains subdivided into subgrains. Diffraction indicated both randomly oriented fine grains and large grains subdivided into subgrains. The critical shear stress of Ag was found to be τ_cr_ = 60 MPa [90], while a direct calculation from the hardness measurements gives τ_cr_ ≈ 150 MPa. The saturation hardness of the thin surface layers of Ag is 780 MPa ± 120 MPa, which is 2.8 times higher than its virgin hardness. High hydrostatic compression prevents crack formation. As the depth of deformation increases, the grain size increases as well, with elongated shear bands forming and displaying varying aspect ratios. Moreover, a significant number of pores are present at a depth of 1 μm in boundary grains and in triple joints (shown by arrows). There is a tendency for the shear bands to tilt approximately 20° relative to the friction surface. The number of dislocations within the grains is limited, and the dislocation boundaries of the lamellae show a wide range of misorientation angles. Grain boundaries can act as both sources and sinks for dislocations in the deformation of nanocrystalline metals and alloys [91]. The high hardness of Ag’s surface layers under friction is likely due to the combined strengthening effects of deformation twinning and dislocation sliding.

### 3.4. Deformation Twinning and Dynamic Recovery in the Transition to Unstable Friction

**Figure 3:**
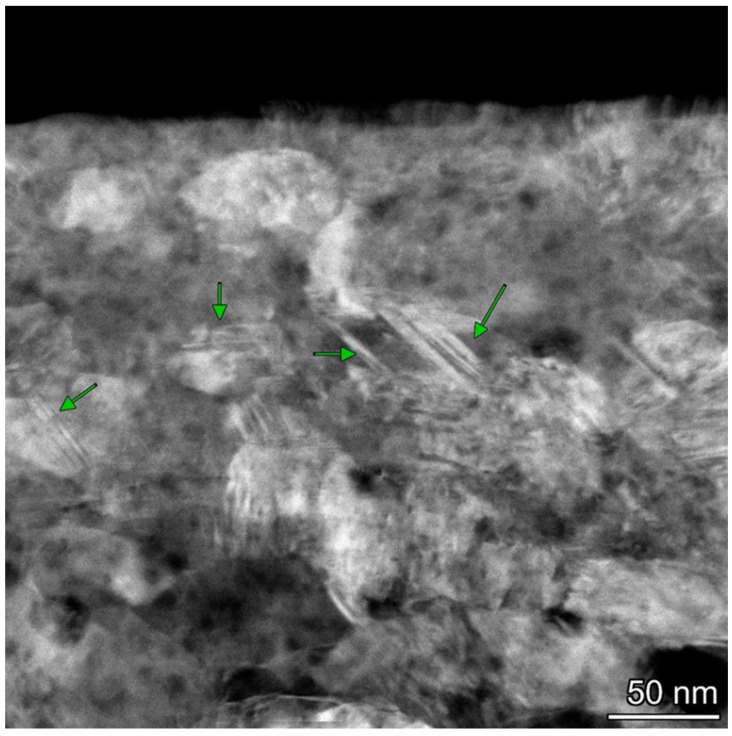
A STEM image of the top surface layers with a large number of deformation twins. The green arrows show the grains with twins.

Twins are formed from Shockley partials emitted from grain boundaries [19,20,21,92,93,94,95,96,97,98,99,100]. Twin generation is mainly influenced by the material’s stacking fault energy [92,99]. A relationship between twinning stress and SFE was proposed by Meyers et al. [19]:

(4) $$\sigma_{T}=K^{*}\;\times \;\sqrt{\frac{\gamma_{SF}}{G\cdot b}}$$

where *G* is the shear modulus, *b* is the Burger’s vector, and K* is a fitting parameter, with K* = 6 GPa for Cu [19]. The stress for twin formation in Ag is calculated to be σ_tw_ = 846 MPa. If the microhardness of Ag’s surface layers after friction is 780 MPa, then the applied stress is approximately 780/3 = 260 MPa, significantly lower than what Meyers’ model predicts for twin formation. However, a more realistic value of the critical stress for twin formation in Ag (σ_tw_ = 160 MPa) was proposed by Zhu et al. [101]. Given that σ_ap_ > σ_tw_, these results are more consistent with the actual findings. The discrepancy in the calculated parameters may also stem from the complexity of determining the fitting parameter K*. We believe that twin generation is a more complex phenomenon than the K* value used. In our view, the grain boundaries of Ag with low SFE serve as primary dislocation sources. The microstructure of Ag’s top surface layers after steady-state friction, characterized by the formation of a significant volume fraction of twin bundles, intersecting twins, and dislocations, is shown in Figure 3.

TEM images reveal numerous narrow parallel lines of stacking faults or nanotwins, as well as twin bundles. The size of grains with nanotwins ranges from 10 to 50 nm, with a twin spacing of 1–2 nm. Notably, twins were generated within the coarse-grain structure (d ~30 μm). The varying locations of twins in the nanostructure make it difficult to determine whether stacking faults were formed through partial dislocation emission or perfect dislocation dissociation. Some bundles of nanotwins traverse entire grains, while others are transformed into twin boundaries (shown by arrows). It is suggested that the cross-slip mechanism is responsible for twin formation at nanograin boundaries [98,99]. Different nanotwin shapes were observed in the TEM images of nano-interiors [16]. These twin boundaries act as barriers to dislocation motion [92,93], significantly enhancing the work hardening of Ag’s surface layers during friction. Several bundles of nanotwins pass through entire grains, while some transform into twin boundaries. The formation of these numerous twin boundaries leads to the refinement of Ag’s upper surface layers [99].

**Figure 4:**
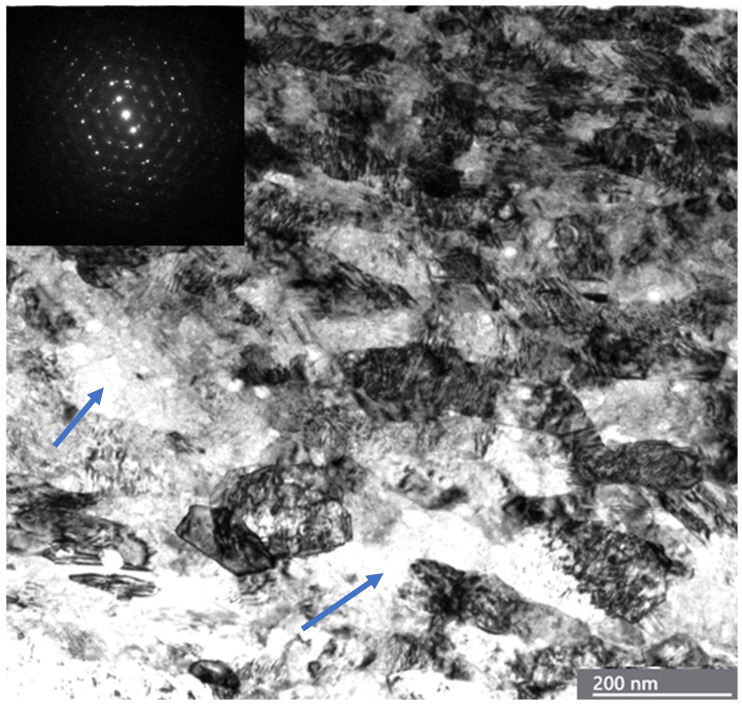
A TEM bright-field image of strong refined top surface layers in the transition to unstable friction. The inset shows the diffraction pattern. Grown grains are shown by arrows.

**Figure 5:**
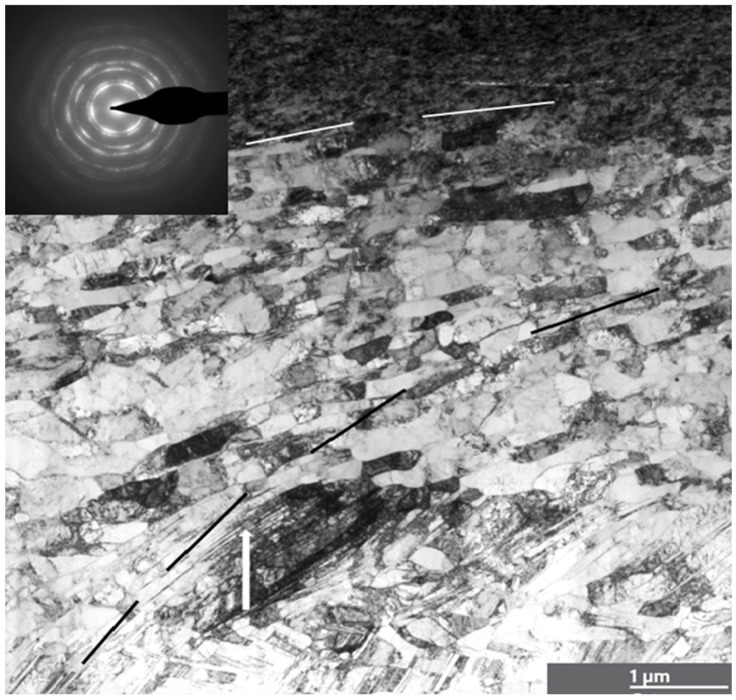
A TEM bright-field image of strong shearing of surface layers resulting from increased temperature and contact pressure. The white and black lines demonstrate strong shearing of ASBs under friction in an unstable state. The twins are shown by the arrow.

As contact pressure and temperature increase, the COF rises slightly (Figure 1 for Ag), representing phase IV: the onset of instability, similar to necking in monotonic or cyclic loading, is shown in Figure 4. Under severe plastic deformation (SPD), adiabatic shear bands (ASBs) are suppressed by the hydrostatic compression of the top surface layers, and the dissolution of twins is expected to result in the formation of new grains. The TEM bright-field image (Figure 4) shows the top surface layers in the range of instability. As the COF increases from 0.12 to 0.18 and the temperature rises to 125 °C (T_t_/T_m_ = 0.18, T_t_-test temperature), the number of twins decreases substantially. An average grain size of ~100 nm is observed. The corresponding SAED pattern indicates that the nanograins are uniformly distributed in the surface layers. Our observations also demonstrate grain coarsening within the refined structure, with grain growth between the refined grains (indicated by arrows). Based on a model predicting recrystallization in single-phase materials [102,103,104,105], dynamic recrystallization (DRX) occurs. This model incorporates incubation time, critical strain, and critical temperature, along with the effect of strain-induced boundary migration. We begin with the concepts of critical strain and temperature for recrystallization. The nucleation of new subgrains between refined cells and deformation twins may be related to stored energy within the refined cells. Subgrain growth kinetics are driven by the competition between the driving force and subgrain mobility. At the macroscale, DRX occurs due to the balance between work hardening and softening, with softening becoming dominant as critical strain (ε_c_) or recrystallization temperature is reached. A TEM image of the sublayers after friction at maximum load (F_N_ = 260 N), COF = 0.18, and T = 125 °C (transition to instability) is shown in Figure 5. Ultra-fine top surface layers are subjected to SPD due to deformation twinning, dislocation sliding, elevated temperature, and high hydrostatic pressure. As the deformation depth increases (>1 μm), adiabatic elongated shear bands (ASBs) are observed. The directions of strong shearing bands are indicated by white and black lines.

**Figure 6:**
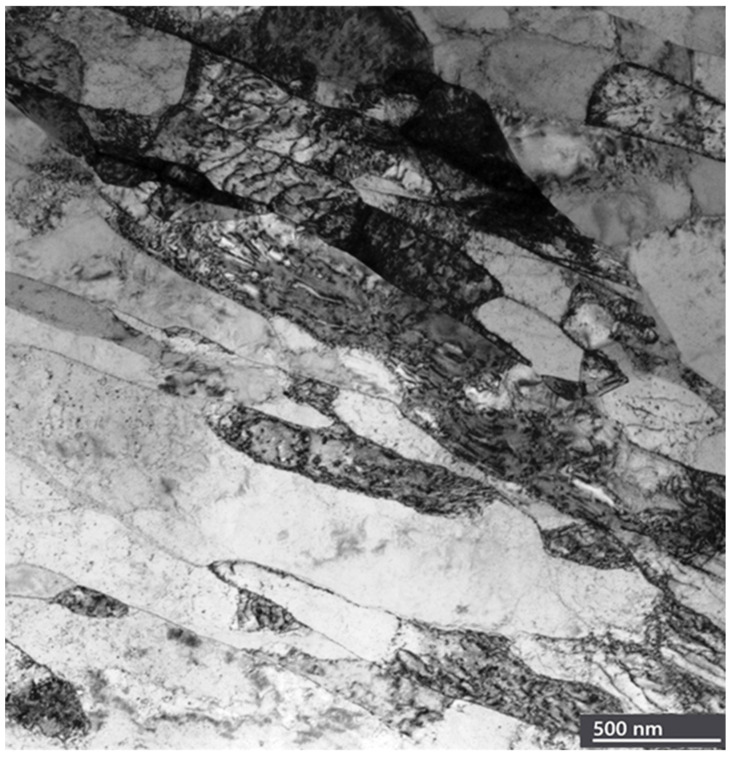
A TEM bright-field image of the details of plastic deformation in ASBs and the development of recrystallization.

Softening at the macroscale increases with depth, corresponding to decreased stress and strain. Ag’s hardness decreases significantly with deformation depth during friction [86,87]. At the mesoscale, grain width varies from 0.1 μm to more than 10 μm, with an average length-to-width ratio of about 3. The twins disappear from the ASB microstructure, although some twin bands can still be seen at minimal deformation in the bottom part of the TEM image (indicated by an arrow). The corresponding selected-area electron diffraction (SAED) pattern in the inset shows large misorientations between subgrains in most areas. Figure 5 shows a STEM image indicating the strong shearing of surface layers resulting from increased temperature and contact pressure. The arrows demonstrate DRX grains. The pores are clearly seen in the substructure layers. An increasing shear tilt was observed as ASB instability and friction intensified. The initial and final ASB curvatures are shown with lines, and it is expected that the curvature of ASBs is associated with the wear track curvature. The increasing curvature may be related to the reduction in deformation resistance in the dislocation structure of the ASBs (Figure 5). Adiabatic shear bands are shown in Figure 6.

Forest dislocations move toward the grain boundaries, which act as sinks for these dislocations or promote dislocation pile-ups. The intense DRX process is associated with minimal applied and slip resistance stresses in the lower regions of ASBs. The microstructures in the upper and lower parts of the ASBs differ significantly: in the upper part, small-sized microbands, elongated dislocation cells, and refined grains (1–2 μm) are observed, while the lower part shows clear signs of DRX. Grain growth is evident inside the shear bands. The dislocation density is substantially lower in the bottom part of ASBs than in the upper part. Meanwhile, microstructural softening, such as DRX, may promote adiabatic shear localization, with DRX preceding ASB formation [106,107,108,109,110,111]. The ASBs in Ag sublayers may form after the plastic deformation of highly deformed cell structures, similar to materials with low SFE. The driving forces for grain boundary movement within ASBs likely arise from local stress differences between adjacent large and small grains. DRX is evident in the lower part of ASBs, where shear stress decreases rapidly, reducing localized plastic deformation and facilitating DRX growth (Figure 5). Although temperature is a key parameter in thermodynamic processes, the adiabatic temperature has only a minor effect on the onset of shear localization [45,46]. TEM and STEM images clearly show the curvature of ASBs aligned with the friction direction in the highly deformed surface layers of Ag. Increased ASB curvature may be related to minimal work hardening and resistance to slip in the bottom part, where plastic deformation is limited. Furthermore, dynamic recrystallization may result from subgrain rotation recrystallization [104]. The grain growth rate is a key factor influencing DRX kinetics and evaluating grain growth in gradient nanograined structures. The average grain sizes in two sections were compared using TEM and STEM images. At a depth of 3.3 μm, the average grain sizes were 390 nm and 500 nm in the steady state and transition to instability, respectively. At a depth of 6.3 μm, these parameters were 590 nm and 720 nm, respectively. The ratio between the grain sizes is 1.3 in the steady state and 1.44 in the unstable state. It is seen that DRX occurs with the depth of the samples. However, the grain sizes become larger as the temperature and load increase in the transition to instability. The grain sizes are ~33 nm and ~700 nm at depths of ~300 nm and ~6000 nm, respectively.

## 4. Discussion

### 4.1. Analysis of Crystal Plasticity Under Friction of Ag and Ni

**Figure 7:**
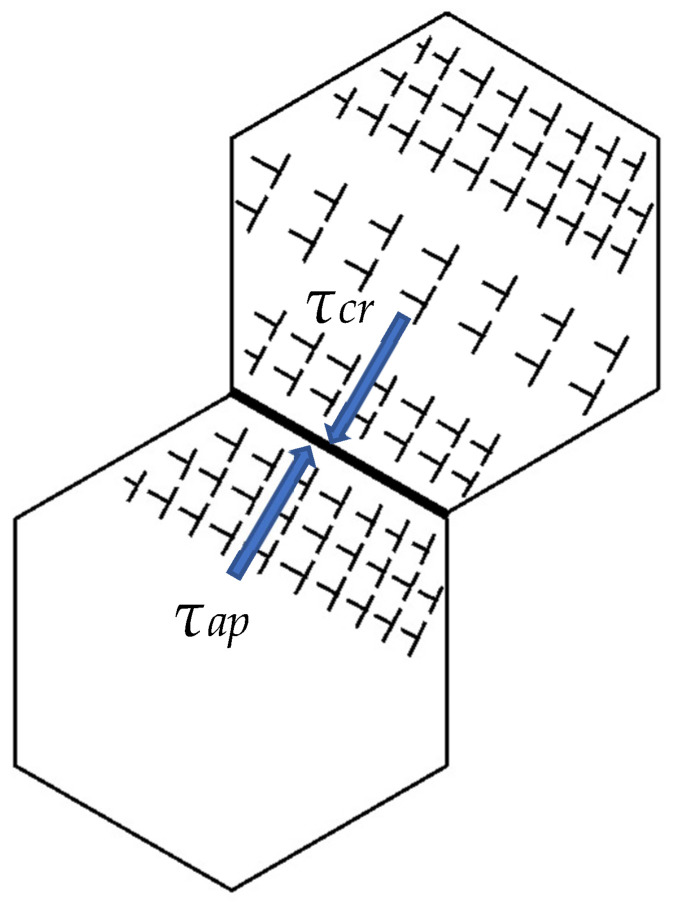
The model of the interaction between the structural elements when the applied stress (τ_ap_) associated with the external loading opposes the deformation resistance (τ_cr_) of the internal dislocation structure. The blue arrow, τ_ap_, indicates the effect of the external applied shear stress. The blue arrow, τ_cr_, shows internal shear stress associated with deformation resistance to applied stress.

According to Newton’s third law, every action (force) in nature has an opposite reaction. In the steady friction state, the storage shear stress due to loading is equal to the deformation resistance stress. In the transition to instability, the shear stress acting on the pile-up should be larger than the critical shear stress. The plastic shear strain rate in the twinning-induced plasticity of Ag (low SFE) under friction, similar to the plastic behavior of TWIP steel [112,113,114], will be studied first. TWIP steel’s constitutive equations will be used to analyze Ag’s plastic deformation under friction in the steady state and in the transition to instability (quick rise in the friction coefficient). The constitutive model of the strain rate and dislocation evolution (Ag) is considered for the first time in terms of friction and wear. The equation for the shear strain rate ${\dot{\gamma }}^{\alpha }$ of the slip system *α* can be presented in the following form [112,114]:

(5) $${\dot{\gamma }}^{\alpha }={\dot{\gamma }}_{0}{\left|\frac{\tau^{\alpha }}{\tau_{cr}^{\alpha }}\right|}^{\frac{1}{m}}sign(\tau^{\alpha })exp(-\frac{Q}{k_{b}T}),$$

where ${\dot{\gamma }}^{\alpha }$ is the reference shear strain rate, *τ^α^* is the resolved shear stress of slip system *α*, $\tau_{cr}^{\alpha }$ is the deformation resistance of slip system *α*, *m* is the strain rate sensitivity exponent, and *Q* is the activation energy. The model of dislocation evolution in the grain structure when the critical shear stress opposes the applied shear during friction is shown in Figure 7. In a state of balance, the applied shear stresses τ^α^ are close to the deformation resistance [87]. Therefore, the state of balance in steady friction should be preserved, τ^α^ ≈ τ^α^_cr_, and the strain rate is close to ${\dot{\gamma }}^{\alpha }$, indicating the dominant elastic contact in the interface. It is expected that the effect of Q/k_B_T is limited in the case of a low homologous temperature, T/T_m_ ≤ 0.3. Slip hardening can be expressed as

(6) $$\tau_{cr}^{\alpha }=\tau_{0}^{\alpha }+\tau_{f}^{\alpha }+\tau_{tw}^{\alpha }+\tau_{b}^{\alpha }$$

where $\tau_{0}^{\alpha }$ is the lattice friction stress, $\tau_{f}^{\alpha }$ is related to forest dislocations, $\tau_{tw}^{\alpha }$ is the twin stress (in materials with low SFE) instead of τ^α^_Gr_, and $\tau_{b}^{\alpha }$ is the back stress.

During the friction of gradient nanograined structures, the lattice stress constitutes a small part of the severe shear stress and can be excluded. Hence, the deformation resistance of FCC metals with low SFE values includes the effects of $\tau_{f}^{\alpha }$, twin stress $\tau_{tw}^{\alpha }$, and back stress $\tau_{b}^{\alpha }$ and can be represented as

(7) $$\tau_{cr}^{\alpha }=\tau_{f}^{\alpha }+\tau_{tw}^{\alpha }+\tau_{b}^{\alpha }$$

We write the equation of the forest dislocation stress according to Taylor’s law [115]:

(8) $$\tau_{f}^{\alpha }=\alpha_{0}G\cdot b\sqrt{\rho_{SSD}}$$

where *α*_0_ = 0.3 is a dimensionless constant (typically 0.2–0.5). *G* is the shear modulus for Ag, G = 30 GPa, and *b* is the Burger’s vector, b = 0.286 nm; $\rho_{SSD}$ is the density of statistically stored dislocations, $\rho_{SSD}$ ≈ 10^14^ m^−2^. Hence, the friction stress ≈ 26 MPa.

Twin formation in TWIP steels is related to SFE, and the critical twinning stress can be described as [114]

(9) $$\tau_{tw}^{\alpha }=\tau_{0}^{tw}+\frac{\gamma_{SF}}{b_{p}},$$

where *τ*_0_^tw^ is the controllable parameter, *γ_SF_* is the SFE value, and *b_p_* is the Burger’s vector of the partial dislocation. The magnitude of the Burger’s vector of a Shockley partial dislocation for FCC metals [88,89] such as silver is b_p_ = *a*/√6, where *a* is the lattice parameter, *a* = 0.406 nm, b_p_ = 0.167 nm [116], γ_SF Ag_ = 16 mJm^−2^ [88,89], and $\tau_{0}^{tw}$ = 100 MPa (TWIP steel) [114]. For the first time, we calculated $\tau_{0}^{tw}$ for Ag as 50 MPa. The deformation resistance of twins increases significantly with dislocation sliding, which enhances the hardening of surface layers during friction, $\tau_{tw}^{\alpha }$~146 MPa.

Meyers et al. [116] estimated the magnitude of τ_tw_ using the Hall–Petch relationship:

(10) $$\tau_{tw}=\tau_{tw}^{0}+k_{T}d^{-1/2}$$

where *k_T_* is material parameter refers to twinning, k_T_ for Cu ≈ 0.7 MN/m^3/2^ [87], d is grain size, and, in this case, *τ_tw_*_Ag_ ≈ 54 MPa. Then, the critical twinning stress for twin formation is [54]

(11) $$\tau_{tw\_cr}=\frac{\gamma_{SF}}{3b_{s}}+\frac{{3Gb}_{s}}{L_{0}},$$

where b_s_ is the Burger’s vector of the Shockley partial, b_s_ = 0.167 nm, and *L*_0_ is the width of a twin embryo, *L*_0_ = 260 nm [95]. From here, the critical twinning stress $\tau_{tw\_cr}\;$ ≈ 90 MPa. The average value of the results obtained with the four measurement methods is $\tau_{tw\_Ag}$ ≈ 150 MPa.

The long-range back stress, τ_b_, induced by deformation twinning, depends on the number of piled-up dislocations [117,118]. The back stress in the nanocrystalline structure is substantially increased, with grain sizes of tens of nanometers. Back stress is affected by the number of dislocations piled up in front of obstacles. They can be stopped when the back stress reaches a critical value, or further dislocations can be emitted in dynamic recovery [116,117,118]. The back stress can be represented in the following form:

(12) $$\tau_{b}=\frac{Gb}{\Lambda }\times \;N^{\alpha }(1-\frac{N^{\alpha }}{N^{*}}),$$

where Λ is the geometrical length scale of the microstructure, 1/*Λ* = 1/*d +* 1/*t_l_* [91]. The thicknesses of twin lamellae [99] are *t_l_* = 50 nm and *Λ*~20 nm. It is worth noting that the size of the pile-ups at the onset of twinning is much smaller than the size of the grains [19].

The factor $1-\frac{N^{\alpha }}{N^{*}}$ refers to the screening effect of back stress. In the first experiment approximation, we calculated the number of dislocations in a pile-up, N^α^ = 2. Hence, the screening effect of back stress is 80%. In fact, deformation twin boundaries are powerful barriers to the transmission of dislocations [90]. The magnitude of N* is driven by the dominant mechanism of relaxation at the boundary. N* is the saturated number of piled-up dislocations for TWIP steel: N* = 8.2 [112] and N* = 20 [114]. In our preliminary experiment with Ag, the number was N* ≈ 10. The critical value $\tau_{b}^{cr}$ (N = N*) indicates that the effect of back stress is diminished under conditions of dynamic recovery. In other words, dynamic recovery is determined by the loss of back stress in the back stress model [116,117,118]. The back stresses become proportional to the total stress for sufficiently large plastic strains. The value of τ_b_ when N = 2, N* = 10, and Λ = 20 nm is calculated to be τ_b_ ≈ 700 MPa. Therefore, the critical shear stress is

(13) $$\tau_{cr}^{\alpha }=\tau_{f}^{\alpha }+\tau_{tw}^{\alpha }+\tau_{b}^{\alpha }=26+150+700=900\;MPa$$

It should be noted that the critical stress at the tip of the pile-up at the onset of failure for Cu is ~1 GPa [119]. Nevertheless, the compressive yield stress of Cu with a grain d = 20 nm is 850 MPa [120].

In the framework of this study, we propose, for the first time, a simplified model of the macroscale applied stress obtained under the conditions of the steady friction state as follows [121,122]:

(14) $$\tau_{ap}=\frac{H}{3\sqrt{3}}\;=\frac{K\mu F_{N}}{A_{r}}$$

where K is the coefficient of similitude [117], which relates the macroscale friction stress state to the mesoscale plastic deformation of the microstructure; μ is the coefficient of friction; F_N_ is the normal friction force; and $A_{r}$ is the real contact area. The relationship τ_ap_ ≈ τ_b_ stated in a steady friction state can be expressed as

(15) $$\frac{K\mu F_{N}}{A_{r}}=K\mu H=\tau_{b}=700\;MPa$$

If the COF in the steady friction state of Ag is 0.1 and hardness H = 780 MPa, then the coefficient of similitude K should be ≈6. The values of the similitude coefficient found in the literature vary from K ≈ 5 to K ≈ 10 [117]. For instance, the similitude coefficient for copper polycrystals is found to be 7.45 [117]. Nevertheless, for cell sizes, the values of K seem to be underestimated because of an inadequate definition of the characteristic distance [117]. This equation will allow the external parameters of loading to be linked with the internal parameters of deformation microstructure at the mesoscale. In our future research, we will focus on the details of a macroscopic model of contact interaction, taking into account geometrical and loading parameters, and stress/strain deformation parameters.

It is expected that the transition to instability can be achieved when τ^α^_ap_ ≥ τ_b_. However, the average hardness of Ag after friction in the unstable region is H = 800 MPa, and thus, the applied shear stress = 800/3√3 ≈ 155 MPa. Nevertheless, if we assume that the hardness is a macroscale parameter and apply, in this case, the coefficient of similitude (K ≈ 5–10) [117], τ_cr_ can vary in the range τ_cr_ ≈ 775–1550 MPa. When N = N*/2, the maximum shear flow stress (*τ_cr_*) and critical strain (ε_cr_) can be calculated as in [92]:

(16) $$\tau_{cr}=\frac{Gb}{d}\cdot \frac{N^{*}}{4},$$

where *G* is the shear modulus, *G* = 30 GPa; b is the Burger’s vector, b = 0.286 nm; and d is grain size, d = 33 nm. According to calculations, $\tau_{cr}$ = 650 MPa ≈ 700 MPa (back stress), and the critical strain is

(17) $$\varepsilon_{cr}=\frac{N^{*}b}{\lambda }\cdot ln2$$

In our experiment, λ = 400 nm (fitting parameter) (λ = 316.5 nm for TWIP steel [112,114] and λ = 413 nm for Cu [87]). The critical strain is ε_cr_ = 5 × 10^−3^ s^−1^. The limited low critical strain can probably be explained by the high magnitude of back stress. The flux of dislocations in the boundary can be described as follows [94]:

(18) $$\frac{dN}{d\varepsilon }=\frac{\lambda }{b}\left(1-\frac{N}{N^{*}}\right)\approx 840\;s^{-1}$$

Clearly, when N = N*, the flux of dislocations at the boundary is fixed, and a crack can initiate. Based on the estimation of τ_f_, τ_tw_, and τ_b_, the critical deformation resistance can be calculated: τ_cr_ ≈ 562 MPa.

Based on the hardening–softening equation first proposed by Kocks, Mecking, and Estrin [122,123,124], the dislocation density during friction of Ag was evaluated as [90]

(19) $$\frac{d\rho }{d\gamma }=k_{1}\sqrt{\rho }-k_{2}\rho +k_{3}\cdot \frac{1}{b}d\left(1-\frac{N}{N^{*}}\right),$$

where the coefficients *k*_1_, *k*_2_, and *k*_3_ have been obtained from [48] for Cu: *k*_1_ = 125 × 10^6^ m^−1^, *k*_2_ = 4.1, and *k*_3_ = 4.4. All other parameters in Equation (12) correspond to Ag after friction in the steady state, where *ρ* = 4.4 × 10^14^ m^−2^, d = 33 nm, b = 0.286 nm, N = 2, N* = 10, and d*ρ*/dγ ≈ 3.7 × 10^17^ m^−2^. In the case of UFG materials, the general assumption for CG materials, d*ρ*/dγ = k_1_√*ρ* − k_2_*ρ* [122,123,124], can be replaced by the following equation:

(20) $$\frac{d\rho }{d\gamma }=\frac{1}{bL}-k_{2}\rho ,$$

where *L* = 1/√*ρ*, and *k*_2_ = 4.1. In this case, d*ρ*/dγ ≈ 7.5 × 10^16^ m^−2^.

The transition to unstable friction is characterized by strong stress relaxation in the shear bands in the bottom part of the deformed Ag sample. The TEM image clearly demonstrates the limited number of deformation twins and a decrease in dislocation density. Hence, in this case, *τ_ap_* > *τ_cr_*; i.e., the deformation resistance of grain boundaries is diminished, and a transition to unstable friction can be achieved. Work hardening of the top surface layers remains high, and surface roughness is low, while strong DRX is observed in the bottom part of the sample. Nevertheless, the wear rate is substantially increased. Generally, understanding the link between the external parameters of contact loading and the internal structural parameters responsible for friction and wear requires further research in tribology. A mesoscale model of the interaction between the structural elements will be developed to understand the co-occurrence of applied and back stresses. In our case, the formation of oxide films on the surface Ni in unstable friction significantly increases the back stress relative to the applied shear stress, thus leading to brittle fracture.

### 4.2. Microstructural Instability

**Figure 8:**
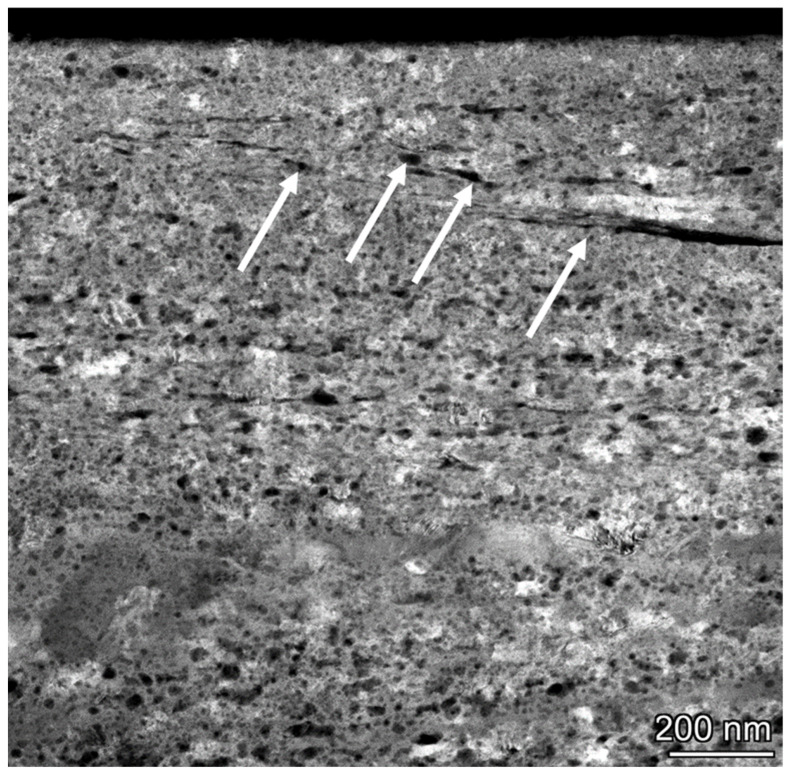
A STEM image of surface layers of Ag with pores joined with cracks, indicating unstable friction (T ~ 160 °C). The arrows point to some of the cracks.

**Figure 9:**
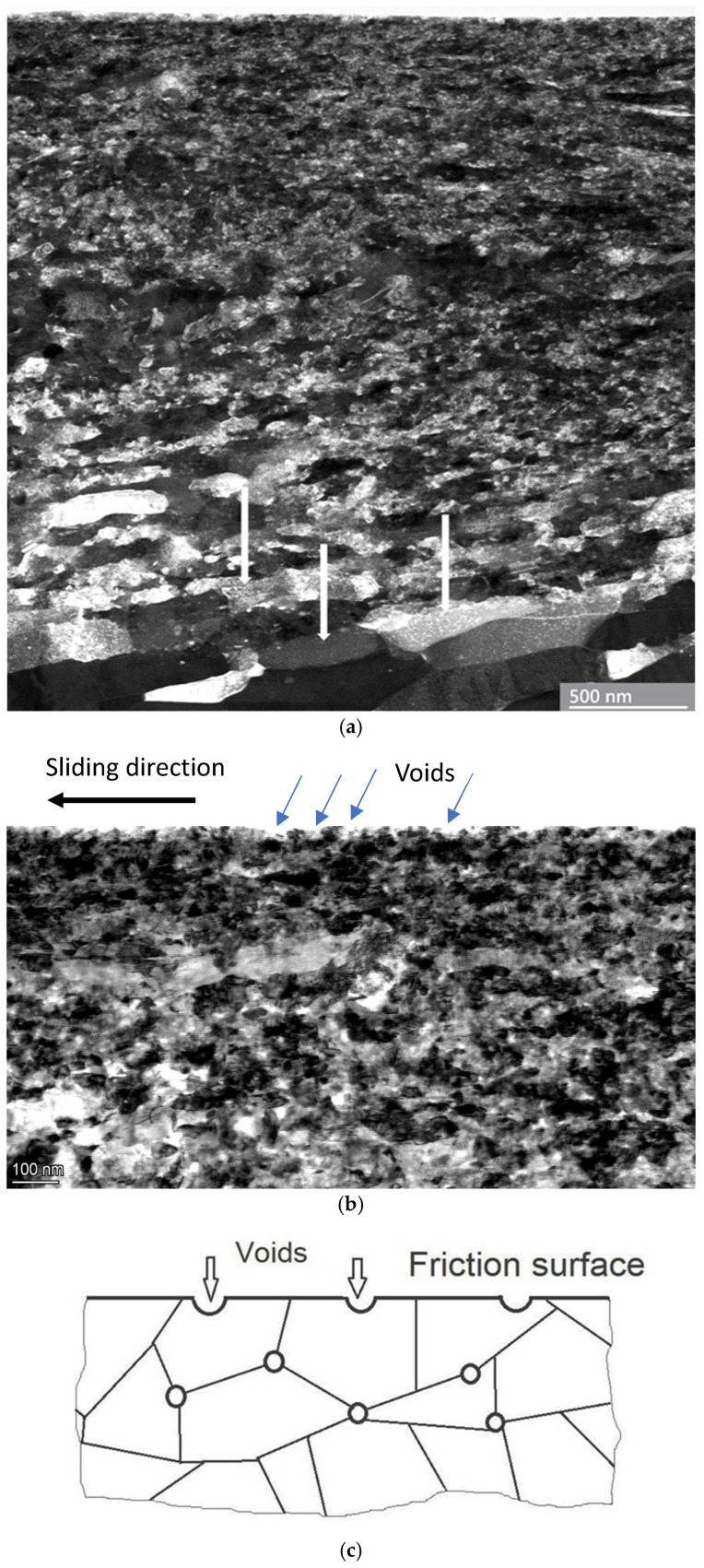
Formation and development of voids and pores during friction of Ag: (**a**) A STEM image of surface layers after severe plastic deformation of Ag and the formation of many pores. The arrows indicate the grains after DRX. (**b**) A TEM bright-field image of the surface layers of Ag, where blue arrows indicate the pores on the contact surface. (**c**) The scheme of a contact surface of Ag with the voids.

After applying friction of Ag in the steady state, a ten-minute experiment with an increasing load from 280 N was conducted, increasing the COF from 0.1 to 0.18 and the temperature to 120 °C. To achieve catastrophic damage to contact surfaces, the load was elevated to 800 N. Under this condition, the COF rose to 0.3 and the temperature to T = 165 °C. However, no seizure or galling phenomena were observed during friction. A STEM image of the pin’s surface after the friction test demonstrates many voids and microcracks from joined voids, as shown in Figure 8. As a result of the low value of SFE for Ag, voids, pores, and microcracks are formed due to the strengthening effects of deformation twinning and dislocation sliding. It should be noted that shearing of the contact surface occurs through voids in the interface, thus decreasing the real contact area and increasing the shear stress significantly (Figure 9a,b). With hydrostatic pressure, the size of the voids increases with the depth of deformation. The upper part of the friction surface with 20–50 nm voids is shown in Figure 9b,c.

Importantly, the nanocrystalline structure and hardness of the top surface layers were preserved. The phase analysis does not reveal the oxide phases. Meanwhile, the surface is relatively smooth without deep grooves, characterizing scuffing and seizure. Plastic deformation in Ag under unstable friction is limited, owing to the interaction of deformation twins and dislocation slip at high hydrostatic compression and relatively high measured temperatures, leading to easy shearing through the porous surface area and, finally, to ductile fracture (Figure 9). Ductile fracture is supported by the relaxation of shear stress around the voids. Shearing can be facilitated by an excessive number of pores. The formation of voids in the interface significantly reduces the real contact area and thus increases the contact pressure.

**Figure 10:**
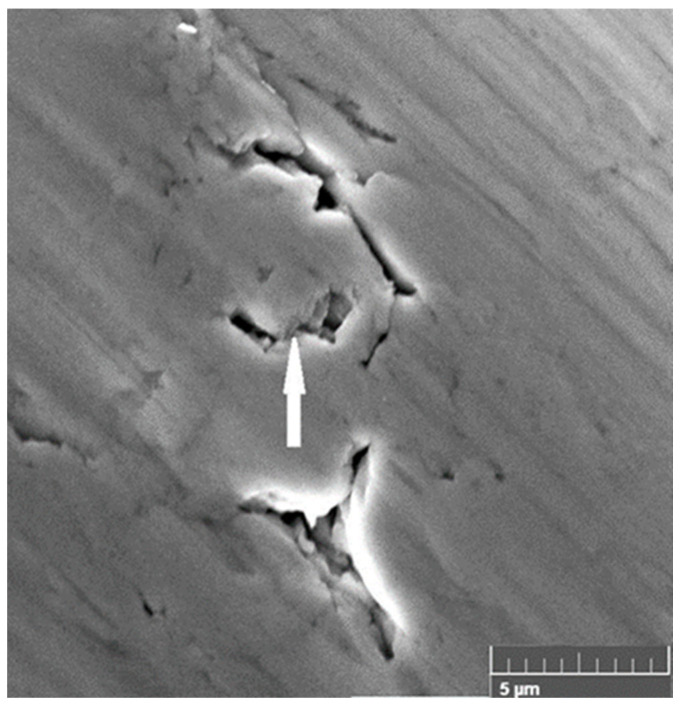
An SEM image of ductile fracture of Ag in the transition to instability. Pores and microcracks are shown by the arrow.

It is emphasized that porosity can mask and/or distort the mechanical properties [125,126]. Meanwhile, the porosity decreases with Young’s modulus [127,128]. The existence of voids varied with the propagation of shear strain: the peak shear strain is distributed around the voids. Further, we observed two linear scales of pore growth: the nanoscale growth of voids and the microscale growth of pores up to the formation of cracks, as observed in Figure 8 and Figure 9. At the nanoscale, the size of the voids varies in the range of ~5–30 nm. Voids are observed at large depths within or near adiabatic shear bands (Figure 9a) [110,111]. The voids coalesce into microscale pores with sizes of up to ≥100 nm. Over time, the length of pores increases along the slip line until microcracks are formed. The SEM image indicates the ductile fracture of Ag with the formation of pores and microcracks on the damaged surface. Subsequently, the preserved refined structure, high number of dislocations, and twins favor the generation of voids along the grain boundaries and triple joints. Importantly, a ductile fracture and a relatively smooth surface, with microcracks of 3–5 μm formed by the interconnection of voids, are observed in Ag (Figure 10).

A high pore density in the wear plane facilitates the delamination of wear particles without the high friction usually observed with catastrophic damage under friction. It is expected that the shear stresses in the interface decrease through stress relaxation as a result of the interaction between deformation twins and dislocations in the surface layers. Similar nano-sized voids with an average diameter of 20 nm were revealed in the grain boundaries of Cu deformed by bending at temperatures between 250 and 400 °C [129]. The formation of voids is explained by the shrinking of voids at grain boundaries after bending. A model of nanovoid growth in deformation twinned materials based on the emission of N dislocations from the void surfaces was described in [130].

Moreover, with an increase in applied stress, the thickness of the twin lamellae is reduced, thus limiting the emission of dislocations and facilitating the formation of pores. At this stage, it was interesting to compare the microstructural stability of Ag under friction with other methods of mechanical tests, for instance, the segregation energies of Ag-based alloys with Fe, Ni, and Co [131]. Recently, a new mechanism of void generation dependent on hydrostatic stress, temperature, and relative vacancy concentration was proposed [132]. With increasing contact pressure, the residual hydrostatic stress, temperature, and vacancy concentration are increased, thus decreasing the activation barriers for void nucleation. The predicted value of average void size was ~35 nm [133], which is close to our estimation. As a result of the Gibbs–Duhem expression, it has been shown that Fe, Co, and Ni components in solid silver can carry a significantly higher surface energy. It is worth noting that the surface energy of the Ag alloy exceeds that of pure silver by more than a factor of two. In this instance, an increase in surface energy for the Ag alloy can lead to some effects responsible for deformation resistance in segregated Ag, such as Griffith stress (σ_c_ = √2Eγ_s/_πr, where *r* is flow size, *E* is Young’s modulus, and *γ_s_* is surface energy), the grain size effect (Hall–Petch relation); and supplementary dislocation barrier (Gb/L_s_ + Δγ_s/_b, where Ls is obstacle spacing, G is shear modulus, b is Burger’s vector, and Δγ_s_ is variation in surface energy). Hence, in this way, the segregation of Ag can be estimated within the framework of the general crystal model of deformation resistance. The applied loading parameters, as well as segregation parameters, including the effect of the chemical medium, can be considered as the general constitutive model of crystal plasticity in our future analysis. In fact, the calculation of grain boundary energy, γ_b_, by comparing the boundaries of ideal and defect structures was proposed:

(21) $$\gamma_{b}=\frac{∆E^{defect}-∆E^{ideal}}{2A},$$

where Δ*E^defect^* and Δ*E^ideal^* are the energies of the polycrystal and the ideal single crystal, respectively, and *A^defect^* is the total grain boundary area [134,135].

**Figure 11:**
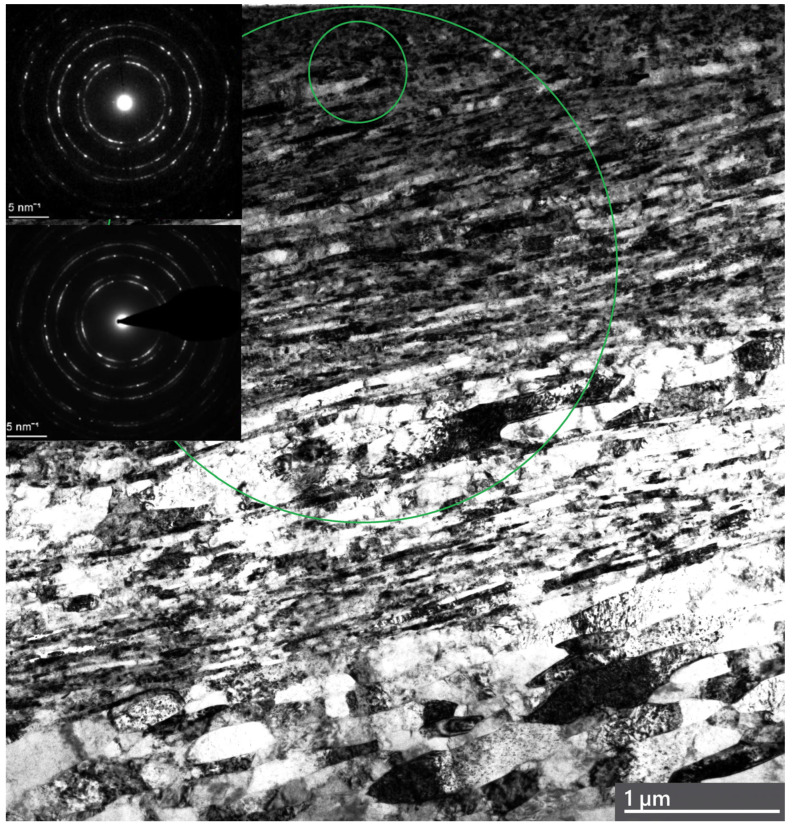
A TEM bright-field image and diffraction patterns of a severely refined structure in the surface layers of Ni in the transition to unstable friction. The upper diffraction pattern taken from the small green circle corresponds to this area. The insert of diffraction in the middle of the image represents diffraction in the big green circle area.

For comparison with the ductile fracture of Ag, we analyzed the brittle fracture of Ni in a similar experiment, where the relaxation of plastic deformation was limited by highly deformed nickel oxide surface layers. The transition to instability can occur when critical shear stress is achieved, and dislocation pile-ups are absent. The transition to unstable friction for Ni was markedly different from that for Ag samples (Figure 11). Thin, elongated shear bands with a thickness of 30–50 nm at a depth of ~300 nm inclined about 20° to the sliding surface are presented in Figure 11. The depth of highly deformed layers is more than 2 μm. At a depth of ~4.5 μm, the grain size is significantly increased. Dynamic recrystallization is observed with a grain size of 500–1000 nm. In the steady friction state, Ni surface layers had a hardness of 2.5 GPa, and the hardness in the unstable region was significantly high at ~6 GPa, which is close to the hardness of NiO [136]. NiO oxide films have high hardness and low fracture toughness. Furthermore, oxide films cannot plastically deform during shearing, resulting in cracks and spalls. Oxide debris can be entrapped between the sliding surfaces by brittle fracture of oxide films. The Ni/NiO metal-oxide interface was the weakest relative to pristine Ni GBs [137]. The oxidized GBs cracked under low tension stress relative to the unoxidized GB range. It is worth noting that the Gibbs energy absorbed per unit area in intergranular brittle fracture is [138]

(22) $$Ga=2G^{surf}-G^{\varphi },$$

where $G^{surf}$ and $G^{\varphi }$ are the Gibbs energies of the free surface and grain boundary, respectively, related to unit area. Further, for simple characterization of grain boundary cohesion, the strengthening/embrittling energy ΔG_SE, I_ was proposed [139]:

(23) $$∆G_{SE,I}=∆G_{I}^{\varphi }-∆G_{I}^{surf}$$

When ΔG_SE, I_ > 0, the segregation of solute *I* leads to decohesion of the interface; when ΔG_SE, I_ < 0, the cohesion of the grain boundary is increased. It is important to emphasize that the presented expressions are similar to those proposed in our experiment.

Let us consider the following possible transitions under friction and conditions of softening/hardening and ductile and brittle fracture:

(a) τ_ap_ > τ_cr_ (τ_cr_ < τ_max_, T ≤ 0.3 T_M_) —hardening;

(b) τ_ap_ > τ_cr_ (τ_cr_ ~ τ_max_, T ≤ 0.5 T_M_) —brittleness;

(c) τ_ap_ = τ_cr_ (τ_cr_ < τ_max_, T ≤ 0.3 T_M_) —steady friction state;

(d) τ_ap_ > τ_cr_ (τ_cr_ = τ_y_, T > 0.5 T_M_) —softening.

It is important to establish that all friction processes reach the steady state, and just after that, instabilities can be captured with the variation in different transition conditions.

The hardness of Ag after friction in the unstable region is 800 MPa. Then, the calculated values of non-dimensional critical shear stress, τ/G, for Ni are about three times larger than those for Ag. Hence, a high density of voids and pores, accompanied by the relaxation of shear stress, led to the ductile fracture of Ag (τ_ap_ > τ_cr_). The surface layers of Ni have high deformation resistance compared to those of Ag, but the applied stress should be higher than τ_cr_ (τ_ap_ > τ_cr_). Generally, the strength–ductility trade-off in applications for studying materials is modeled as follows: τ_app_ > τ_cr_ < τ_max_—strengthening/softening; τ_app_ > τ_cr_, τ_cr_ ≡ τ_max_—brittleness.

**Figure 12:**
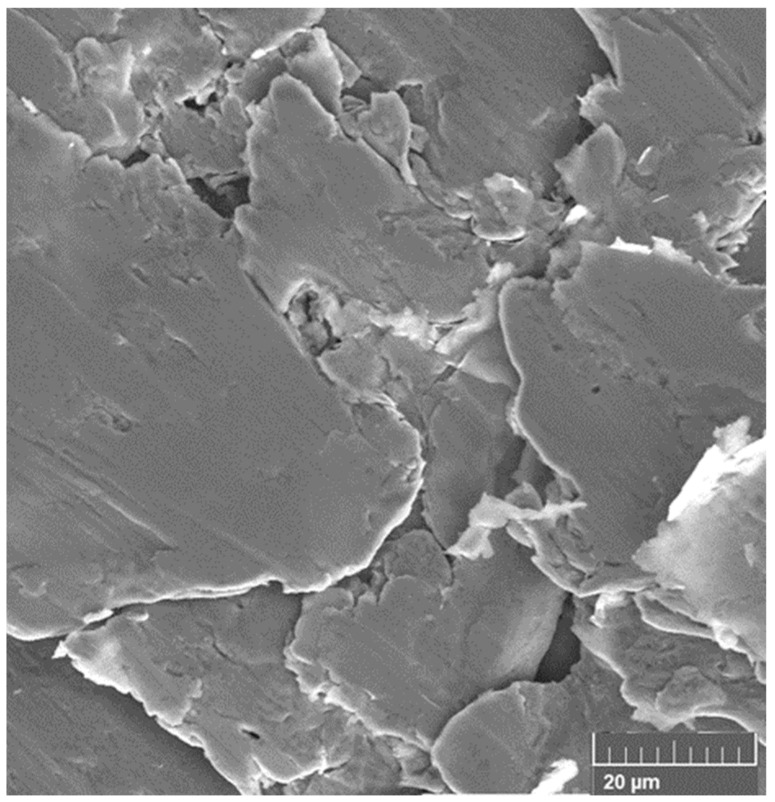
An SEM image of the brittle fracture of the surface layers of NI after friction in the unstable region.

Analysis of the damaged surface of Ni clearly shows the brittle fracture (Figure 12).

### 4.3. Model of Strength–Ductility Trade-Off and Microstructural Instability

The relationship between dislocation pile-ups and cross-grain driving forces must be better understood (see Figure 7). Two strategies to improve the thermal stability of NC materials were developed: a kinetic one, in which GB migration *M* is decreased, and a thermodynamic one, in which the driving force, i.e., GB energy, *G_b_*, is suppressed [103].

Shear stress and temperature can cause dislocation pile-ups to migrate through the grain boundary. Meanwhile, drag dislocations or driving forces may provide deformation resistance on the other side. It is generally assumed that the velocity of dislocations in the grain boundary is given by v = M × P, where M is mobility of dislocations and P is the driving pressure. If, under defined conditions, the mobility is controlled by solute atoms, then the solute drag pressure *P_sol_* can affect the boundary velocity by decreasing the driving pressure as *P* = (*P_d_* − *P_sol_*). Hence, the driving forces provide deformation resistance to dislocation pile-ups formed due to applied stress and temperature. Moreover, the migration of pile-up dislocations at the grain boundary can be considered as follows [103]:

(24) $$M=M_{0}\exp\left(-\frac{Q}{k_{B}T}\right),$$

where *k_B_* is Boltzmann’s constant, *Q* is the apparent activation energy related to the atom-scale thermally activated process. As can be seen, dislocation migration is mainly determined by the activation energy and work surface temperature, and it can depend on the conditions of segregation.

When we consider the steady-state deformation state observed under different loading conditions, such as tension, fatigue, friction, or corrosion, saturated shear stress is achieved. In this case, variations in the mobility and velocity of dislocations in the boundaries are diminished—ΔV~0. However, the driving pressure *P* can be high. As the external loading parameters increase, dislocation pile-ups can interact with dragging dislocations, inevitably leading to effects such as hardening, softening, or brittleness. At this stage, the velocity of migrating dislocations can be represented as [140].

(25) $$\frac{\gamma^{4}\nu_{D}}{k_{B}T}\exp(-\frac{∆G_{m}}{k_{B}T}\cdot \frac{∆G^{\#}}{\Omega })=M\times P,$$

where *γ* is the atomic diameter, *ν_D_* is the Debye frequency, *Ω* is the atomic volume, and Δ*G_m_* and Δ*G^#^* are the migration Gibbs energy and the activation Gibbs energy of segregation, respectively [140].

In the presence of a segregated solute at the grain boundary, its atmosphere applies a force opposite to grain boundary migration, acting as a so-called drag force. Therefore, the driving force for grain boundary migration is reduced by the term (*P_d_* − *P_sol_*). The drag effect of solutes on grain boundary migration has been successfully applied to stabilize nanostructures [141,142]. A nanostructure stability map was developed based on the enthalpies of mixing and segregation as a function of stability, metastability, and instability. In the framework of the regular nanocrystalline solution (RNS) model, a particular alloy system was described by the interaction parameters *w*, which are used to define the solute–solvent bond energies within the crystal (c) and GB regions:

(26a) $$\omega_{c}=E_{c}^{AA}-\frac{E_{c}^{AA}-E_{c}^{BB}}{2}$$

and

(26b) $$\omega_{ab}=E_{ab}^{AB}-\frac{E_{ab}^{AA}-E_{ab}^{BB}}{2},$$

where E is the energy per bond, classified by the superscript representing the solvent (A) or solute (B) and the subscript denoting the bond type. Unfortunately, the described stability problem does not focus on the numerical values of the input parameters, which limit the application of these maps to different applications. In the development of our model, the stability can be considered as a balance of pile-ups due to the effect of dislocation mobility on the applied parameters—temperature, shear modulus, parameters of the solvent and solutes from one side (E_app_), and the dislocation resistance opposing the pile-ups according to the general expression (E_cr_) E_eff_ = E_ap_ − E_cr_. In contrast to our experiment, the relationship between the thermodynamic driving force Δ*G* and the kinetic energy barrier *Q* was analyzed in [83]. Phase transformations and plastic deformations are governed by the kinetic behavior of atoms activated by thermodynamic forces. The thermo-kinetic correlation and generalized stability (*GS*) during plastic deformation were considered:

(27) $$∆G=∆G_{\sigma }-∆G_{\tau }\propto \;\sigma_{b}-CGb\rho^{1/2},$$

where Δ*G* is defined as the difference between the driving force exerted by the applied stress (Δ*G_σ_*) and the glide resistance due to the mutual interaction of dislocations (Δ*G_τ_*), *b* is the Burger’s vector, *σ_b_* is the flow stress, *G* is the shear modulus, *ρ* the dislocation density, and *C* is the geometrical factor. It should be noted that this equation correlates directly with our analysis results. In our experiments, *τ_eff_ = τ_ap_* − *τ_cr_*, where *τ_eff_* is the effective shear stress resulting from the interaction between the applied and resistance stresses. Moreover, we consider the steady state, *τ_eff_* ~ 0 and *τ_ap_ ~ τ_cr_*. It is also noteworthy that in our evaluation, τ_cr_ includes different components of plastic deformation, but τ_app_ is related directly to the external loading conditions. The generalized stability, Δ, is determined as Δ *= Q*/*Q_y_* − Δ*G*/Δ*G_y_*. If the authors considered GS in the yield point, then in our experiments, we analyzed the hardening/softening stability.

## 5. Conclusions

In this study, the deformed microstructures of Ag and Ni, metals with low and high values of stacking fault energy (SFE), were examined, focusing on the generation and annihilation of dislocations. In addition, the constitutive model of plastic deformation was investigated, along with the effects of twinning and dynamic recovery on friction both in the steady state and during the transition to instability. TEM, STEM, and SEM analyses of deformed surface layers were performed to analyze Ag and Ni friction properties. In this study, grain boundary hardening and softening, microstructural stability, strength, ductility, and brittleness were evaluated. A thermo-kinetic approach was used to analyze microstructural stability and instability. The following conclusions are drawn:

(1) The effect of dislocation pile-ups responsible for the generation or annihilation of dislocations during friction was considered. Ag’s top surface layer exhibits steady-state friction under the formation of twin bundles, intersecting twins, and dislocations. With increasing deformation depths (>1 μm), adiabatic elongated shear bands (ASBs) were observed. The intense dynamic recrystallization (DRX) process was associated with minimal slip resistance and applied stresses in the lower regions of ASBs. The mechanisms of microstructural stability and friction instability and the theoretical models of dislocation generation and annihilation in ultrafine-grained (UFG) or nanocrystalline (NC) FCC metals were investigated, particularly in the context of plastic deformation and failure development under friction.
(2) The transition to unstable friction was estimated. Plastic deformation in Ag’s surface layers was limited due to the interaction of deformation twins and dislocation slip in a hydrostatic compression field. The damaged surface of Ag exhibited numerous pores, reducing the contact area and significantly increasing the shear stress. The brittle fracture of Ni represents a catastrophic failure associated with the formation of super-hard nickel oxide. It is worth noting that the Gibbs energy absorbed per unit area in intergranular brittle fractures is similar to the effective shear stress considered in our work.
(3) Deformation resistance of the mesoscale dislocation structure was compared to the applied shear stress at the macroscale. The coefficient of similitude (K) has been introduced in this work to compare plastic deformation at different scales. The maximum calculated values of shear flow stress (τ*) and critical strain (ε*) were compared with experimental results. The model of the strength–ductility trade-off and microstructural instability is considered. The interaction between the migration of dislocation pile-ups and the driving forces applied to the grain boundaries was estimated. It is suggested that driving forces provide deformation resistance to dislocation pile-ups formed due to the applied stress and temperature during friction. The phase transformation and plastic deformation in the literature are attributed to the thermodynamically induced kinetics of atoms, which is similar to our effective shear stress, τ_eff_ = τ_ap_ − τ_cr._
(4) Nanostructure stabilization through the addition of a polycrystalline element (solute) to the crystal interiors in order to reduce the free energy of grain boundary interfaces was investigated. Nanocrystalline GS can be stabilized via solute segregation, especially at high temperatures. It is suggested that further refinement of these complex systems is key to the development of new strengthening materials. A method for suppressing strain localization was proposed, involving selective doping for grain resistance. The thermodynamic driving force and kinetic energy barrier involved in strengthening, brittleness, or annealing under plastic deformation and phase formation in alloys and composite materials were examined.

## Figures and Tables

**Table 1:** The load (F_N_), coefficient of friction, virgin (Hi) and final (Hf) hardness, stacking fault energy, grain diameter d, shear modulus (G), and homologous temperature under friction in the steady state.

Material	Load F_N_, N	COF	H_i_, GPa	H_f_, GPa	γ_SF_, mJm^−2^ [88,89]	d, nm (XRD)	T/T_m_	G, GPa
Ag	220 ± 20	0.10 ± 0.02	0.27 ± 0.01	0.78 ± 0.12	16	33 ± 3	0.26	30
Ni	1820 ± 200	0.08 ± 0.01	0.78 ± 0.03	2.60 ± 0.25	125	160 ± 5	0.31	75

## Data Availability

The original contributions presented in this study are included in the article. Further inquiries can be directed to the corresponding author.

## Nomenclature

*2y*	dislocation length	*j*	dislocation flux
*a*	lattice parameter	*k*	Archard wear coefficient
*A_r_*	real contact area	$k_{B}$	Boltzmann constant
*A^defect^*	grain boundary area	*K*	coefficient of similitude
*ASB*	adiabatic shear bands	*K**	fitting parameter
*α*	slip system	*L*	obstacle spacing
*α*_0_	dimensionless constant	$L_{0}$	width of twin embryo
*b*	magnitude of the Burger’s vector	µ	coefficient of friction (COF)
$b_{p}$	Burger’s vector of a partial dislocation	Λ	geometrical length scale of the microstructure
*b_s_*	Burger’s vector of the Shockley partial	*ν_D_*	Debye frequency
*d*	size of grain	m	strain rate sensitivity exponent
DRX	dynamic recrystallization	M	migration of pile-up dislocations
$∆E^{ideal}$	energy of the single crystal	$N^{\alpha }$	number of dislocations
$∆E^{defect}$	energy of the polycrystal	$N^{*}$	saturated number of piled-up dislocations
*E_seg_*	segregation energy,	*n*	strain hardening exponent
*ε_cr_*	critical strain	*P*	driving pressure
*H*	hardness	*ρ*	dislocation density
*F*	Gibbs energy absorbed per unit area	*ρ_SSD_*	density of statistically stored dislocations
*F_N_*	normal friction force	*Q*	activation energy
*G*	shear modulus	*σ_tw_*	twinning stress
GBs	grain boundaries	*σ_ap_*	applied stress
$∆G^{\#}$	activation Gibbs energy	$\sigma^{surf}$	Gibbs energies of the free surface
ΔG_chem_	strain energy	$\sigma^{\varphi }$	Gibbs energies of grain boundary
$∆G_{m}$	migration Gibbs energy	*t_l_*	thickness of twin lamellas
ΔG_strain_	interface energy	*t*	time
ΔG	interface energy	*T*	temperature
ΔG _SE, I_	strengthening/embrittling energy	*T*_c_	recrystallization temperature
*λ*	fitting parameter	*T_t_*	test temperature
*λ_p_*	mean free path	*T*_m_	temperature of melting
*γ*	atomic diameter	*τ*	shear strength
${\dot{\gamma }}^{\alpha }$	shear strain rate	$\tau_{0}^{\alpha }$	lattice friction stress
*γ_b_*	grain boundary energy	$\tau_{cr}^{\alpha }$	critical shear stress of slip system α
*γ_SF_*	stacking fault energy	$\tau_{b}^{\alpha }$	internal backstress
$\tau_{q}$	shear stress concentration	*V*	wearing volume
$\tau_{f}^{\alpha }$	stress of forest dislocations	*Ω*	atomic volume
$\tau_{tw}^{\alpha }$	twin stress	*w*	interaction parameters
$\tau_{tw\_cr}$	critical twinning stress	*W*	dimensional wear coefficient
$\tau_{0}^{tw}$	controllable parameter	UFG	ultrafine-grained crystals
